# Effects of Vitamin E on Cognitive Performance during Ageing and in Alzheimer’s Disease

**DOI:** 10.3390/nu6125453

**Published:** 2014-11-28

**Authors:** Giorgio La Fata, Peter Weber, M. Hasan Mohajeri

**Affiliations:** DSM Nutritional Products Ltd., R & D Human Nutrition and Health, P.O. Box 2676, CH-4002 Basel, Switzerland; E-Mails: peter.weber@dsm.com (P.W.); hasan.mohajeri@dsm.com (M.H.M.)

**Keywords:** α-tocopherol, antioxidant, oxidative stress, brain ageing, AD

## Abstract

Vitamin E is an important antioxidant that primarily protects cells from damage associated with oxidative stress caused by free radicals. The brain is highly susceptible to oxidative stress, which increases during ageing and is considered a major contributor to neurodegeneration. High plasma vitamin E levels were repeatedly associated with better cognitive performance. Due to its antioxidant properties, the ability of vitamin E to prevent or delay cognitive decline has been tested in clinical trials in both ageing population and Alzheimer’s disease (AD) patients. The difficulty in performing precise and uniform human studies is mostly responsible for the inconsistent outcomes reported in the literature. Therefore, the benefit of vitamin E as a treatment for neurodegenerative disorders is still under debate. In this review, we focus on those studies that mostly have contributed to clarifying the exclusive function of vitamin E in relation to brain ageing and AD.

## 1. Introduction

Free radicals are molecules containing a reactive unpaired electron. In biological models, the majority of free radicals contain an atom of oxygen and, therefore, are called reactive oxygen species (ROS) [1]. ROS are mainly produced in mitochondria and represent important regulators of cell signaling and cell cycle progression [2]. At high concentrations, however, ROS are detrimental and responsible for the biological damage that compromises cellular functions [3]. In 1956, Harman postulated the free radical theory of ageing [4], whereby ageing is considered a progressive, inevitable process partially related to the accumulation of oxidative damage in biomolecules [1]. The original view of Harman, even if revolutionary for that time, was probably too simplistic in that ROS may not just function stochastically [5]. There is now evidence indicating that ROS also function as specific signaling molecules, and the increased protein oxidative damage during the aging process may be a targeted, rather than a stochastic phenomenon [5,6]. Although cellular damage is still widely considered the general cause of ageing [7,8,9,10], nine candidate hallmarks have been recently proposed to contribute most to the aging process [10]. In the central nervous system (CNS), this molecular damage is also postulated to be responsible for neurodegeneration and, consequentially, for the onset of pathological conditions typical of old age, such as AD and dementia [1].

**Figure 1:**
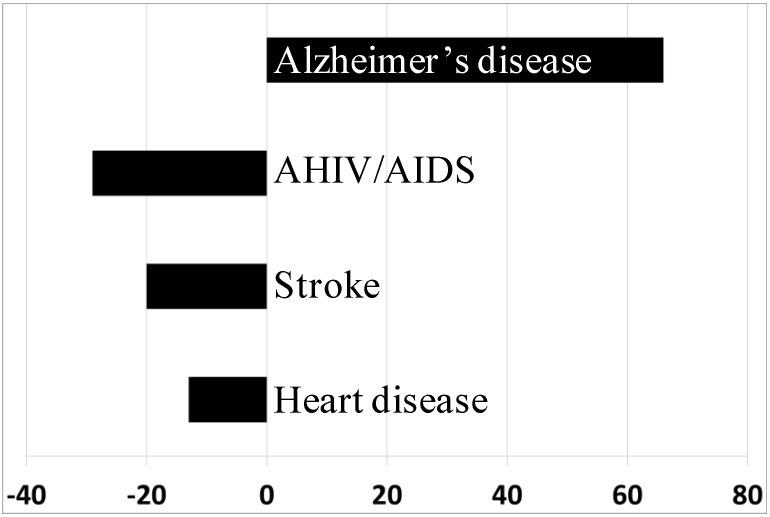
Changes in disease-related deaths (%). Numbers of deaths caused by HIV, stroke and heart disease-declined substantially between 2000 and 2008. Within the same time period, AD-related deaths increased more than 65%. Adapted from [42].

AD is a chronic, progressive neurodegenerative disorder characterized by a functional decline in memory and other cognitive capabilities [11,12]. AD prevalence is age dependent, and it is the most common form of dementia, accounting for 60%–80% of dementia cases [11]. While the numbers of deaths due to HIV, stroke and heart disease have dropped consistently in the last decade (Figure 1), the corresponding incidence of AD has increased dramatically [13]. Medical estimations performed in 2008 discovered that people with AD and dementia cost 19-times more to the society when compared with age-matched people without dementia [11]. In the U.S. alone, costs associated with AD were estimated to be around $203 billion in 2013 [11]. Moreover, as world population is ageing, incidence is increasing. In 2005, the worldwide incidence of dementia was approximately 24 million, and 4.6 million new cases were estimated to be diagnosed every year. By 2050, the global prevalence of dementia cases is predicted to quadruple [14].

Considering that AD and related dementia are caused by irreversible neuronal damage, current available treatments are inadequate [13]. It is, however, conceivable that preventing or delaying AD-related pathological conditions represents the most valid alternative to treatment. For instance, delaying the onset of the AD clinical phase by just one year can reduce disease prevalence by 25% [15] with enormous positive economic and social impacts for society.

The prevention of the cognitive decline associated with AD and dementia can be influenced by a number of factors, including nutrition [12,13]. Current epidemiological data highlight the beneficial functions of specific micronutrients (in particular vitamins) to ease these debilitating pathologies [13,15]. Recent studies underpin the positive role of nutrition in preventing AD-related disabilities. It was demonstrated that diet supplementation with vitamins B and E positively affects the pathological hallmarks observed in patients with mild and moderate AD, including a delay in cognitive decline [16,17]. In addition, docosahexaenoic acid (DHA) supplementation in adults with age-related cognitive decline was shown to improve cognitive health [18]. Similar results were also observed in another study, where higher dietary intake of omega-3 (ω-3) polyunsaturated fatty acid (PUFA) was associated with lower plasma levels of amyloid-beta42 (Aβ42), a profile associated with reduced risk of AD incidence and slower cognitive decline [19].

Vitamin E includes a group of eight structurally-related, lipid-soluble, chain-breaking antioxidants: four tocopherols and four tocotrienols: α (alpha), β (beta), γ (gamma) and δ (delta). α-Tocopherol is the most abundant and bioavailable antioxidant form of vitamin E in human tissues [20,21]. A dietary antioxidant is a substance in food that significantly decreases the adverse effects of reactive species, such as reactive oxygen and nitrogen species, on normal physiological function in humans [22]. These antioxidants can convert free radicals into less reactive compounds and, therefore, protect cellular components that are vital for the correct functioning and survival of complex systems [23].

The importance of vitamin E in the CNS [24,25] was already evident at the beginning of the 20th century when Evans and Burr described paralytic offspring of rats deprived of dietary vitamin E [26]. Several other reports followed, linking vitamin E deficiency with pathologies affecting the motor activity of humans whose symptoms could be reverted by vitamin E supplementation [27,28,29]. Ataxia with vitamin E deficiency (AVED) is an autosomal recessive cerebellar ataxia in humans caused by mutations in the α-tocopherol transfer protein, leading to low levels of serum vitamin E [30,31,32]. Treatment of AVED patients with vitamin E showed clinical improvements, especially in early stages of the disease [33,34]. The neurological importance of vitamin E is also underlined by its association with other brain disorders. Low levels of α-tocopherol in the brain were shown in carriers of the APOE epsilon4 (ɛ4) variant (a significant risk factor for AD) [35], as well as in patients with AD and mild cognitive impairment (MCI) [36,37,38]. Moreover, a reduced risk of developing AD was observed in subjects with high plasma levels of vitamin E [39] and following vitamin E intake [40,41]. The scope of this work is to provide an overview of the clinical and epidemiological studies performed to assess the effects of vitamin E on cognitive performance during ageing and in pathological conditions, such as AD.

## 2. Vitamin E and Brain Function

### 2.1. Epidemiological Evidence

The importance of adequate nutrition in support of healthy brain function was already reported in the 1980s, when direct links between nutritional status and cognitive performances were established. In a cohort of 260 healthy people (aged > 60 years), a positive link between cognitive performance and higher nutrient concentrations (folate, vitamin B12, vitamin C and others) in blood plasma was described [43]. The same positive effect was also reported years later in another study: 304 individuals were examined for their cognitive performance in relation to nutrient content and after dietary supplementation with specific vitamins (including vitamin C, E and thiamine) [44]. Both studies reported modest, but significant, improvements in cognitive performance of individuals with a satisfactory nutritional status. Of note, the authors claimed that the associations were modest, probably because both studies enrolled healthy people with no nutrient insufficiencies [44].

The aforementioned observations were confirmed by another study examining memory performance in 442 healthy individuals (age > 65 years) [45]. Higher plasma concentrations of ascorbic acid and beta-carotene were, in this case, associated with improved memory performance [45]. Two years later, a survey involving a multiethnic population in the United States reported that memory performances were linked to vitamin E and not to vitamin C and beta-carotene. Precisely, poor memory performance was consistently evident when low plasma levels of vitamin E were measured (serum vitamin E levels normalized per unit of cholesterol) [46]. Further evidence emphasizing the positive role of vitamin E in brain function is the finding that high levels of α-tocopherol (and vitamin A) are found in plasma of cognitively normal centenarians [47], possibly contributing to the protection against oxidative stress and, thereby, to their cognitive function. Finally, another convincing positive association between vitamin E status (measured as the serum α-tocopherol concentrations and α-tocopherol/cholesterol ratio) and cognitive function in an aging population was found in a study of 34 men and 84 women (aged between 65 and 91 years) who were free of significant cognitive impairments. Subjects with vitamin E intakes less than 50% of the recommended daily intake (RDI) were shown to perform cognitively worse than those with a higher intake level [48].

These observations are in line with the results of another report [49] that linked consumption of vitamin C and E supplements with better cognitive performances. Grodstein and collaborators analyzed the cognitive performances of a very large number of participants (approximately 15 thousand women aged 70–79 years) after vitamin E and C supplement intake for approximately 20 years. The results showed that users of vitamin E and C supplements have better cognitive performances than non-users and that in longest term users of these supplements (>10 years), the effect of consuming vitamin E and C was cognitively equivalent to being 1.5 years younger. Importantly, while the intake of vitamin E and C alone showed little evidence of improving the cognitive capacities of the users, their combination was necessary to obtain significant effects [49]. Clear benefits were also described when comparing women with low dietary intake of α-tocopherol to those with high intake. The difference, in this case, was equivalent to being cognitively two years younger [49].

The Cache County Study is a prospective study of elderly residents of Cache County (Utah, USA). Previous data analyses from this study revealed a reduced AD risk for participants taking antioxidant vitamin supplements or non-steroidal anti-inflammatory drugs (NSAIDs), as well as a reduced cognitive decline in individuals eating food rich in antioxidants [50]. Because the possible synergic effect of antioxidants and NSAIDs was not investigated, Fotuhi and collaborators analyzed the available data to assess the potential combined effects of vitamins E or C and NSAIDs on cognitive decline [50]. Participants of this study were considered users of vitamin E if they reported to take it at a minimum of four times a week and for a month or longer. Users were also considered those taking, with a similar frequency, multivitamin preparations containing at least 400 international units (IU) of vitamin E. In total, data from 3376 participants were included in this analysis, and cognitive decline was assessed by the Modified Mini-Mental State examination evaluated up to three times over an eight-year period [50]. At baseline, users of vitamin E (or C) performed better than non-users, but a positive association with better cognitive performance was observed only after combined use of vitamins E and C and NSAIDs [50].

Both epidemiological and experimental studies have demonstrated that a diet rich in fruit and vegetables has a beneficial effect on cognitive function [51]. These food groups are rich in antioxidants that act as free radical scavengers that protect the brain from neuronal damage [51]. The *Supplementation en Vitamines et Mineraux Antioxydants* 2 (SU.VI.MAX2) is an observational study conducted to investigate the effect of nutrition (especially fruit and vegetables) on the quality of aging [51]. In the analysis, 2533 healthy participants of the SU.VI.MAX2 study were included to investigate the possible link between the intake of specific nutrients (among them, vitamin E) from food (fruit and vegetables, in particular) and ageing (nutrients from supplementation were excluded) [51]. Participants were invited to provide a 24-h dietary record every two months (six times/year) for a total period of two years that was successively analyzed in combination with the cognitive assessments already available in the SU.VI.MAX2 study. Interestingly, the nutrient intake model provided by the authors, showed that vitamin E was positively associated with better cognitive performance [51], as evaluated by verbal memory and executive function scores [51].

Morris and colleagues monitored the cognitive changes of 2889 healthy people (aged 65 to 102 years) over a period of three years in the presence of high and low antioxidant consumption [52]. Four different cognitive tests revealed a reduced cognitive decline per year in individuals with higher vitamin E intake (users) (obtained by diet and from supplements) when compared to non-users (low vitamin E intake) [52]. This difference was lost once the non-users started to consume food with high vitamin E levels [52]. Conversely, few effects were observed when carotene, vitamins C and A were analyzed [52]. In a similar study, vitamin E intake was associated with improved cognitive performance and with a decreased risk of developing AD [41,53].

The Women’s Health Study (WHS) is a double-blinded, placebo-controlled, randomized trial of vitamin E supplementation with a 10-year duration [54]. Originally, this study was designed to monitor the preventive effects of vitamin E and aspirin on cardiovascular diseases and cancer [54]. In 1998 (5.6 years after randomization), a sub-study was started to evaluate the cognitive performance of 6,377 elderly healthy women randomized to receive vitamin E (α-tocopherol acetate) 600 IU/every other day with a follow-up period of four years. The evaluation of the cognitive performance was conducted by telephone interviews through an adaptation of the Mini Mental State Examination (MMSE). The authors reported that compared to the placebo group (*n* = 3,193), the vitamin E group (*n* = 3184) did not have a lower risk of substantial cognitive decline [54]. Of note, this study showed fewer adverse cognitive changes when the vitamin E group was compared to the placebo group with dietary intake of vitamin E below the median of 6.1 mg/day [54]. Additional favorable effects were also observed, including parameters related to exercise and diabetes measures [54].

Taken together, these epidemiological studies demonstrate that consumption of specific micronutrients, including vitamin E, is linked to improved cognitive performance in humans. Therefore, vitamin E was examined in AD, a pathological condition characterized by cognitive decline.

To monitor the preventive function that specific micronutrients may have on the AD onset, 5395 healthy individuals (mean age, 55 years) were monitored for a period of six years. Out of 5395, 197 participants developed dementia, of whom, 146 had AD [55]. The authors described a lower risk of developing AD in individuals consuming food with a high content of vitamin E and C (vitamin E >15 mg/day) [55].

The preventive effect of vitamin E with respect to developing AD symptoms was demonstrated in a study of 232 dementia-free subjects aged 80+ years, derived from the Kungsholmen Project [39]. Subjects with high plasma levels of total tocopherols, total tocotrienols or total vitamin E had a reduced risk of developing AD in comparison to persons with lower levels [39]. Similar observations were also reported two years later [56]. A study population derived from the AddNeuroMed cohort, one of the largest cohorts in Europe to identify biomarkers for AD, was used to evaluate the relationship between MCI and AD and the plasma contents of all vitamin E forms in individuals with MCI and AD (521 subjects) [56]. The authors identified an association between low plasma tocopherol and tocotrienol levels and increased odds of MCI and AD [56].

There is ample reason to suspect that ventricular cerebrospinal fluid (vCSF) represents a more adequate compartment to study the brain status than circulating blood [57]. For this reason, Hensley and collaborators measured the vitamin E levels (α and γ tocopherol) in the vCSF of post-mortem AD patients [57]. In agreement with other studies that found lower tocopherol plasma levels among AD patients [58,59,60], the authors found that higher concentrations of vCSF α-tocopherol were associated with better performance in perceptual speed, as well as in lower neuritic plaque density [57]. Conversely, the global cognitive scores did not change in relation to α-tocopherol concentrations [57]. The fact that vCSF and plasma vitamin E level are both reduced in AD patients provides strong evidence of the importance of this vitamin in supporting brain function in healthy subjects.

Epidemiologic Studies of the Elderly (EPSE) is a ten-year prospective study with the aim of describing predictors of mortality and risk factors for chronic diseases and loss of functioning [61]. A sub-group of subjects already enrolled in the EPSE was monitored for ten years, and the probability of developing dementia or AD in relation to specific parameters, including vitamin use, was calculated. Six hundred sixteen persons were included in this secondary analysis, of whom 141 developed dementia [61]. In general, the authors declared that consumption of high doses of vitamin E (and C) was not associated with a delayed development of dementia or AD [61]. Several reasons may have led to this null result. Of note, in this case, the demented population was quite heterogeneous, including 93 people with AD, 30 with vascular dementia (VaD) and 18 with general dementia. Unfortunately, no information regarding the duration and the dose of vitamin E use was available. Finally, only a very small proportion (less than 10%) of the subjects included in this study used these antioxidants.

In addition, consumption of vitamin E or C alone was associated with improved cognitive performance also in another study [62]. It is noteworthy that, even if a significant protective effect for general dementia was demonstrated in men that consumed vitamin E and C supplements together [62], no beneficial effects were detected when the dementia was associated with AD [62]. Unfortunately, an important limitation of this study, with no positive outcome, is the lack of information about dosage and the duration of the supplementation, as well as other confounding factors, such as information about the health status of the participants. By and large, the results reported from these observational studies support a beneficial effect of vitamin E in AD patients.

### 2.2. Intervention Studies

The cognitive impairments and the behavioral symptoms that characterize AD are associated with a loss of cholinergic neurons in the brain and increased oxidative stress [17]. Oxidative stress increases during ageing and represents one possible cause for the onset and progression of AD [1]. For this reason, several strategies for AD treatment have focused on enhancing cholinergic neuronal function or promoting neuroprotective effects through the administration of specific antioxidants [17]. Due to the antioxidant properties of vitamin E and considering the promising results obtained from animal studies [63,64] in preventing neuronal death and delaying ageing, the use of vitamin E to treat patients with AD and other forms of dementia typical of old age has been examined in several clinical trials (Table 1).

**Table 1:** Overview of clinical trials supporting vitamin E supplementation in individuals with AD.

Study details	Supplementation Dose Duration	Primary outcomes Main results Side effect due to vitamin E	Secondary outcomes Main results	Reference
**Study:** Alzheimer’s Disease Cooperative Study	2000 IU/day (1000 IU/twice a day) Duration: 2 years	Time to the occurrence of any of the following end points: death; institutionalization; loss of ability to perform at least two of the three basic activities of daily living: eating, grooming, using the toilet; severe dementia Results: Significant delay in the institutionalization time for the α-tocopherol group when compared with placebo (*p* = 0.003) No significant side effects were found between groups after adjustments for multiple comparisons	Measurement of: cognition; function; behavior; presence or absence of other extrapyramidal signs Results: beneficial effects following α-tocopherol treatment (Blessed Dementia Scale, *p* = 0.004) Less supervision was necessary for the patients treated with α-tocopherol (*p* = 0.021)	[65]
**No. of participants:** Total study: 341 Placebo *n* = 84 α-tocopherol *n* = 85				
**Pathological stage:** AD patients (moderate severity)				
**Average age:** 73 years				
**Study:** Alzheimer’s Disease Cooperative Study	1000 IU/day (first 6 weeks) 2000 IU/day (remaining time) Duration: 3 years	Time to the development of possible or probable AD (starting from MCI) Results: No significant differences in the probability of progression from MCI to AD when the vitamin E group is compared to placebo (*p* = 0.91) Treatment with vitamin E did not produce any unexpected side effects.	Measured parameters: (Mini Mental State Examination ) MMSE; Alzheimer’s Disease Assessment Scale, cognitive subscale (ADAS-cog); (global Clinical Dementia Rating) global CDR; (Alzheimer’s Disease Cooperative Study-Activities of Daily Living) ADCS-ADL Results: No significant differences were observed between vitamin E and placebo group	[66]
**No. of participants:** Total study: 769 Placebo *n* = 259 Vitamin E *n* = 257				
**Pathological stage:** Mild Cognitive Impairment (MCI)				
**Average age:** 72 years				
	800 IU/day Duration: 6 months	Glutathione oxidation Results: Patients with moderate (n = 26) and severe (*n* = 6) dementia have a higher concentration of basal oxidized glutathione (GSSG) level when compared to healthy controls (*n* = 18) (*p* < 0.05) Higher GSSG/reduced glutathione (GSH) ratio for severe (*n* = 6) demented patients when compared to moderated demented patients (*n* = 26) (*p* < 0.05) No side effects mentioned in this study	Measurement of: cognition; function; behavior; presence or absence of other extrapyramidal signs Results: beneficial effects following α-tocopherol treatment (Blessed Dementia Scale, *p* = 0.004) Less supervision was necessary for the patients treated with α-tocopherol (*p* = 0.021)	[67]
**No. of participants:** 33 AD patients Healthy controls *n* = 18 AD placebo *n* = 14 AD vitamin E *n* = 19				
**Pathological stage:** 25 with mild, 26 with moderate and 6 with severe dementia				
**Study:** Trial of Vitamin E and Memantine in Alzheimer’s Disease (TEAM-AD)	2000 IU/day (1000 IU/twice a day) Duration: from 6 months to 4 years	Activities of daily living Results: Over the mean follow-up time of 2.27 years, participants receiving α-tocopherol had significantly slower decline than those receiving placebo (ADCS-ADL, *p* = 0.03) Reduced annual rate of decline in ADLs by 19% when the α-tocopherol group is compared to placebo No vitamin E specific adverse effect observed	Measured parameters: MMSE; ADAS-cog; Neuropsychiatric Inventory (NPI); Caregiver Activity Survey (CAS); Dependence Scale Results: Favorable effect of α-tocopherol considering ADAs-cog and CAS (not statistically significant after adjustments for multiple comparisons)	[17]
**No. of participants:** Total study: 613 AD patients α-tocopherol *n* = 140 Placebo *n* = 140				
**Pathological stage:** Mild to moderate AD				
**Average age:** 79 years				

One of the first randomized controlled trials (RCTs) performed to study the effects of vitamin E on AD pathology treated 341 AD patients (moderate severity) with selegiline and vitamin E (dl-α-tocopherol (Hoffmann-LaRoche, Nutley, NJ, USA) 1000 IU/twice per day) for two years [65]. After vitamin E supplementation (*n* = 85), there was a significant delay in the deterioration of daily life activities, as well as a reduced need for care [65]. The level of α-tocopherol was monitored by measuring serum tocopherol concentrations. Tests for α-tocopherol were considered positive if serum tocopherol levels were 2.0 mg per deciliter (46 µmol per liter) or higher in 75% of the blood samples obtained from a given patient [65]. No improvements of the cognitive test scores were observed in this study, possibly due to the relatively advanced severity of AD in this population at the onset of supplementation. This last point highlights the importance of the “brain status” of the to-be-treated population. The clinical severity of AD may indeed influence the success probability of the intervention, the progression of the disease and the associated cognitive impairments.

The positive results associated with vitamin E supplementation in patients with severe AD [65] prompted investigating if similar or even more beneficial effects could also be obtained in early stages of AD. MCI represents a transitional state that progresses to AD [66]. Previous studies have demonstrated that 10%–15% of people with mild cognitive impairment develop AD within one year [66]. This number reduces to a rate of 1%–2% among normal elderly people [66]. Petersen and collaborators enrolled 769 subjects (average age 72 years) with amnestic MCI from the Alzheimer’s Disease Cooperative Study [66]. A three-year study involving vitamin E supplementation of 2000 IU/day was conducted. This study failed to demonstrate any significant difference in the probability of progression from MCI to AD after vitamin E supplementation [66]. Of the 769 participants, 214 had progression to dementia, among which 212 were classified as having possible or probable AD with an overall rate of progression of 16% [66]. No unexpected side effects were observed following vitamin E treatment [66].

Twenty-four of the recruiting sites involved in the Alzheimer’s Disease Cooperative Study [66] also decided to participate in a magnetic resonance imaging (MRI) sub-study [68], as MRI measurements may be a useful diagnostic tool to identify the brain atrophy that succeeds a pathological condition. Atrophy rates are greater in both AD and MCI subjects, and in addition, it was shown that MCI subjects with greater hippocampal atrophy rates were more likely to convert to AD [68]. The purpose of this study was to evaluate the effects of vitamin E treatment on brain atrophy using MRI. Brain atrophy rates were determined by annual percentage change. One hundred thirty one subjects were included, and the size of their hippocampus, entorhinal cortex, whole brain and ventricular volumes were analyzed [68]. A trend, which did not reach significance, towards lower atrophy rates was observed in those groups treated with vitamin E and donepezil [68]. In agreement with published data, the authors found a greater rate of brain atrophy in those patients who converted to AD from MCI, as well as in APOE ɛ4 carriers whose conversion rate to AD was more likely to occur [68].

The possibility that vitamin E could have beneficial effects on the cognitive properties of AD patients was investigated mechanistically [67]. A reliable indicator of vitamin E activity measured in this study was the blood oxidized glutathione level. Fifty seven AD patients (mild, moderate and severe dementia) were recruited, and 33 completed the study. Interestingly, it was found that people treated with vitamin E (800 IU/day for six months) were able to maintain their cognitive status (and even performed slightly better) over the study period only if lower blood oxidized glutathione levels were detected. Conversely, when vitamin E was not effective as an antioxidant, the authors observed a worsening of the cognitive performances [67]. This study highlights the anti-oxidative properties of vitamin E as the mechanism of action and as a therapeutic approach against AD pathology [67].

The Trial of Vitamin E and Memantine in Alzheimer’s Disease (TEAM-AD) is an RCT, designed to assess the efficacy of α-tocopherol, memantine or their combination in delaying clinical progression of AD in patients taking an acetylcholinesterase inhibitor [17]. This study started in 2007, was completed in 2012 and included 613 participants (mainly men, mean age of around 79 years). One hundred fifty two randomized patients with mild to moderate AD (assessed by MMSE) were supplemented with vitamin E (dl-α-tocopherol acetate) and compared to 152 randomized placebo-treated patients. The duration of the supplementation ranged from six months to four years, making this study one of the largest and longest treatment trials in patients with mild to moderate AD [17]. The authors found that 2000 IU/day of α-tocopherol significantly delayed the clinical progression of AD symptoms and decreased the caregiver burden associated with it [17], confirming data generated in another multicenter study that treated severe AD patients with α-tocopherol [65]. Serum concentration of vitamin E at baseline was measured prior to randomization and in annual assessments. Cut points of 1.3-fold or greater increases in α-tocopherol were associated with a reasonable level of medication adherence [17]. In addition, favorable effects (but not statistically significant) were associated with α-tocopherol treatment when memory and language properties were considered, as well as the time necessary for the caregivers to assist the patients. Moreover, the authors found no safety concerns associated with 2000 IU/day vitamin E supplementation when compared to the control group [17].

## 3. Possible Mechanisms beyond the Antioxidant Function

The cognitive decline observed during ageing and in AD is associated with increased oxidative stress [1], which may be partially responsible for the time-dependent accumulation of cellular damage [10], which ultimately leads to neuronal death and neurodegenerative disorders. Being a potent antioxidant vitamin and essential to life, vitamin E has stimulated researchers to investigate how it affects the cognitive decline that is observed in pathological conditions and during normal ageing. However, the biological relevance of vitamin E goes beyond antioxidant activity. Recently, new functions were associated with vitamin E, including its role in signaling, membrane fluidity and gene regulation.

Vitamin E regulates the activity of multiple signal transduction enzymes whose activities consequentially affect gene expression [23]. For example, α-tocopherol inhibits the activation of the protein kinase C (PKC) [69,70] by preventing its phosphorylation [71], as well as its localization to the membrane [72]. Moreover α-tocopherol was shown to enhance the protein phosphatase 2A (PP2A) activity, an enzyme that is implicated in AD-pathophysiology (for a review, see [73]). Other enzymatic activities are also modified by vitamin E, with consequential effects on cell proliferation [74] inflammation [75] and cellular adhesion [76] (for comprehensive reviews, see [23,69]). Microarray data from rodent studies [69] showed that vitamin E also regulates the expression of specific genes related to oxidative stress, muscles structure, cholesterol metabolism, amongst others (see [69] and the references therein for details).

Vitamin E deprivation experiments performed in rats demonstrated that in the hippocampus, the expression of a number of genes linked to the onset and progression of AD was vitamin E responsive [77]. The identified genes were important regulators of hormone metabolism, apoptosis, growth factors, neurotransmission and amyloid-beta metabolism [77]. Of note, the hippocampus of rats deficient in vitamin E showed a decreased expression of the APP binding protein 1 [77], whose activity is to bind and stabilize APP, the precursor of the Aβ fragments, which are associated with AD.

Additionally, animal experiments showed that low α-tocopherol levels in the brain induce downregulation of genes involved in myelination and synaptogenesis, neuronal vesicle transport and in glial functions [78]. These data strongly support the hypothesis that optimal coverage of the organism with vitamin E is an important determinant of healthy brain functions throughout life.

A recent study has also described a protective role for vitamin E against AD pathology [79]. Combined *in vitro* and *in vivo* experiments confirmed a mechanism by which vitamin E protects against the formation of the major AD biomarker, hyper-phosphorylated tau. Vitamin E in this case was able to prevent the activation of p38MAPK, whose activity is essential for phosphorylation of neuronal tau molecules [79].

The beneficial effect of vitamin E is also evident in models of Smith–Lemli–Opitz Syndrome (SLOS) [80]. SLOS, caused by mutations in the gene encoding the last enzyme in cholesterol biosynthesis, 7-dehydrocholesterol (7-DHC) reductase, is characterized by phenotypic malformations, as well as cognitive impairments and autistic-like behaviors [80]. The authors reported that vitamin E supplementation was sufficient to inhibit the peroxidation of 7-dehydrocholesterol (a hallmark of SLOS) and that feeding a vitamin E-enriched diet to pregnant females led to a decrease in oxysterol formation in brain and liver tissues of the newborn animals in this model.

Despite the great importance of these studies in completing the knowledge about vitamin E functions, they are mainly obtained *in vitro* or *in vivo* using animal models mimicking the human vitamin E deficiencies. In the literature, there are very few data reporting non-oxidative functions of vitamin E in human studies and brain, in particular.

## 4. Discussion

The increased oxidative stress that occurs during ageing represents a possible cause of AD onset and progression [46,81,82,83]. Therefore, use of the potent antioxidant, vitamin E, has been investigated as a treatment to delay the onset or the progression of this pathology, as well as to ameliorate the cognitive decline naturally occurring during ageing.

Despite the high number of studies performed to assess the antioxidant effects in pathological conditions and during ageing, only a few tested exclusively the vitamin E effects in humans. Of all the studies cited in this review, only four were designed to test the specific effect of vitamin E in treating, preventing or delaying AD [17,65,66,67] . Therefore, only these works will be discussed further.

A recent large study demonstrated that vitamin E supplementation significantly delayed the clinical progression of AD symptoms in patients with mild and moderate AD [17]. These data corroborated older results [65], where a reduced functional decline in patients with moderately severe AD was observed following vitamin E supplementation [65]. These studies suggest that supplementation of vitamin E (2000 IU/day) may be sufficient to delay the functional decline observed in AD pathology at different stages of its progression.

Of note, in both cases, no significant differences in cognitive performance were observed when the placebo group was compared to the vitamin E supplemented group, although a trend for the beneficial effects of vitamin E was observed [17]. A possible explanation provided by the authors relates to the stage of the pathology. The AD pathology may have been too advanced in the enrolled patients, such that no differences were appreciated when cognitive performance was measured. Indeed, the authors conclude that perhaps functional and occupational measures of cognitive capacity are better indicators of disease progression than psychometric measures [65]. Additionally, the effects of vitamin E on cognitive performance may have been masked by acetylcholine esterase inhibitor therapy, the standard therapy for patients in these trials, or by the presence of varying confounding factors, such as other diseases. Indeed, in another study [54], it was observed that vitamin E treatment was (cognitively) beneficial among women without diabetes, but not among women with diabetes [54]. Moreover, other important information, such as dose and duration of supplementation, as well as the number of participants using the supplements, is necessary to evaluate a specific vitamin E effect [61].

The phase that precedes the clinical AD stage is characterized by the presence of MCI. In 2005, a study was performed to monitor the effect of vitamin E supplementation during early stages of AD [66]. The authors claimed that no beneficial effects were associated with vitamin E administration to patients with MCI [66]. In particular, the probability of progressing from MCI to AD after vitamin E supplementation was not affected. Therefore, the authors concluded that vitamin E supplementation did not delay the progression of the AD pathology at early stages [66].

A possible reason to justify such inconsistencies is given by the difficulty to perform precise and uniform studies. AD is a multi-faceted, progressive neurodegenerative disorder with different levels of severity. In the studies by Dysken *et al.* [17] and Sano *et al.* [65], the criteria to recruit the participants were the presence of possible or probable AD (mild and moderate severity), while in the study by Petersen *et al.*, the subjects enrolled had amnestic mild cognitive impairment [66]. In all of the above cases, 2000 IU/day of vitamin E were administered to each participant (for approximately three years), but in a later study [66], the initial administered dose was 1000 IU/day and, later (six weeks after the beginning of the study), 1000 IU twice daily [66]. Moreover, subjects enrolled by Dysken and collaborators were under medication (taking an acetylcholinesterase inhibitor (AChEI) [17]), while no medications were reported by Sano *et al.* [65] or by Petersen *et al.* [66]. Finally, Dysken *et al.* [17] and Sano *et al.* [65] measured the capacity to carry out daily life activities, while in the other study, the primary outcome was the probability of progressing from MCI to AD [66]. In summary, varying outcome measures are tested in different studies, which may not be directly comparable to each other.

Beneficial effects (measured by the absence of cognitive decline over time) following vitamin E supplementation were observed in another AD cohort [67]. Importantly, in this case, the cognitive performance of the participants was constant over time (and even slightly improved) only when the vitamin E antioxidant activity was confirmed to be effective. Conversely, deleterious effects (pronounced loss of cognitive abilities) were present when no enhanced antioxidant levels were detected [67]. To explain the lack of effect in the non-responders, the authors speculated about a possible pro-oxidant activity of vitamin E [67]. Another possible explanation could be that the anti-oxidant defense of non-responders does not utilize vitamin E as well as the responders. In this case, the measurement of vitamin E levels in the plasma of the participants would have clarified this point. This explanation and the positive effects of vitamin E supplementations in the responders strongly lend support to the proposal that vitamin E supplementation would be important in limiting the cognitive loss observed in AD patients.

As mentioned before, the difficulty in performing precise and uniform studies accounts for the varying results. For example, in the study by Lloret *et al.* [67], only 57 AD patients were studied, of which 25 were diagnosed with mild, 26 with moderate and six with severe dementia. Vitamin E supplementation included 800 IU/day, as opposed to 2000 IU/day in the other studies. The supplementation period was six months, while in other studies, supplements were given for approximately two to three years. Finally, the participants enrolled in the study performed by Lloret *et al.* [67] were taking standard anti-cholinesterase drugs, similar to [17], but not in [66] and in [65]. Last, but not least, the primary and secondary measures of these studies were different. All of these factors may result in varying outcomes and may mask the positive effects of supplementation.

An important parameter that can influence the outcomes of such studies relates to the form of administered vitamin E. In studies by Dysken *et al.* [17] and Sano *et al.* [65], the same form of vitamin E (dl-α-tocopherol) was used, and in both cases, the α-tocopherol status of the subjects was measured in the serum. This uniformity contributes to an easy comparison of the two studies. This information, however, was not reported in the other reports reviewed, even when the measurement of the vitamin E levels would have been useful for a comprehensive understanding of the proposed results.

Another factor complicating direct comparison of the outcomes of human data is the baseline level of vitamin E. In Goodwin *et al.* [43] and La Rue *et al.* [44], the authors claimed that the observed effects were modest, because the compared subjects had a similar satisfactory nutritional status, thus a similar concentration of vitamin E at baseline. In healthy people, this parameter is easily influenced by varying vitamin E intake by diet, high or low consumption of vitamins, implying that the subjects enrolled in these studies may have a different oxidative status. This concept becomes even more important when long-term trials are performed and when they include pathological conditions, such as AD. In conclusion, even if the micronutrient content at baseline is usually measured prior to interventional studies, due to its high variability in different populations, it represents another parameter that needs to be considered when multiple interventional studies are compared.

Several epidemiological studies indicate that vitamin E from food sources is more effective at preventing age-related neurodegenerative disorders than dietary supplementation [84]. This idea is supported by the fact that vitamin E from food sources comprises all four tocopherols and four tocotrienols, whose properties and possible functions are different [85]. Although α-tocopherol is the most abundant and bioavailable form of vitamin E in human tissues, it was demonstrated that tocotrienols may be more potent radical scavengers than α-tocopherol under specific experimental conditions [84,86,87]. Taken together, the differences in the results between RCTs and observational studies could be due to the varying chemical forms present in the supplements and in food, as well as their bioavailability. In addition, the combination of nutrients from food, as seen in observational studies, may have interactive and synergic effects on health. Such beneficial effects may be masked or mitigated in supplementation trials.

## 5. Conclusions

The National Health and Nutrition Examination Survey data from 2003 to 2008 show that intakes of vitamins A, C, D, E, K and folate are low in a significant proportion of the elderly population in the U.S. [13]. In Germany, vitamin D and folate appear to be the most critical vitamins in people aged 65 to 80 years, followed by vitamin E and C [13].

This review highlights the importance of adequate vitamin E intake in support of healthy brain function in the elderly.

Most of the epidemiological studies analyzed in this work clearly associate high levels of vitamin E with improved cognitive performance and reduced risk of developing AD. For this reason, vitamin E use has been investigated to ameliorate the cognitive decline naturally occurring during ageing and as a treatment to delay the onset or the progression of AD.

Several RCTs show the beneficial effect of vitamin E supplementation in delaying the functional decline observed during AD progression. The socioeconomic benefits that could be derived from a delay in the need for care for these patients are enormous. Unfortunately, other RCT set-ups failed to associate vitamin E use with a reduced cognitive decline in AD, as well as delayed AD onset. Therefore, more standardized research is needed to identify a clear effect of vitamin E on cognitive decline observed during ageing, as well as during AD progression from early to late phases [88].

Importantly, the studies analyzed here confirmed that vitamin E supplementation (even at a dose of 2000 IU/day for an average of two years) is safe and free of specific side effects in the elderly.

In conclusion, the positive effects obtained in the above-cited RCTs, the relative safety of vitamin E combined with the low cost and the absence of valid alternative treatments for AD, suggest vitamin E as a nutritional compound to promote healthy brain ageing and to delay AD-related functional decline. Further research is required to substantiate the emerging and encouraging evidence related to vitamin E’s effects on brain health.
